# Impacts of Indoor Radon on Health: A Comprehensive Review on Causes, Assessment and Remediation Strategies

**DOI:** 10.3390/ijerph19073929

**Published:** 2022-03-25

**Authors:** Leonel J. R. Nunes, António Curado, Luís C. C. da Graça, Salete Soares, Sérgio Ivan Lopes

**Affiliations:** 1 PROMETHEUS, Unidade de Investigação em Materiais, Energia e Ambiente para a Sustentabilidade, Instituto Politécnico de Viana do Castelo, 4900-347 Viana do Castelo, Portugal; acurado@estg.ipvc.pt; 2 Escola Superior Agrária, Instituto Politécnico de Viana do Castelo, 4990-706 Ponte de Lima, Portugal; 3 Escola Superior de Tecnologia e Gestão, Instituto Politécnico de Viana do Castelo, 4900-348 Viana do Castelo, Portugal; sil@estg.ipvc.pt; 4 UICISA:E, Unidade de Investigação em Ciências da Saúde: Enfermagem, Escola Superior de Saúde, Instituto Politécnico de Viana do Castelo, 4900-347 Viana do Castelo, Portugal; luisgraca@ess.ipvc.pt (L.C.C.d.G.); saletesoares@ess.ipvc.pt (S.S.); 5 ADiT-Lab, Instituto Politécnico de Viana do Castelo, 4900-347 Viana do Castelo, Portugal; 6 Instituto de Telecomunicações (I), Campus Universitário de Santiago, 3810-193 Aveiro, Portugal

**Keywords:** indoor radon, health impacts, indoor radon assessment, indoor radon remediation

## Abstract

Indoor radon exposure is raising concerns due to its impact on health, namely its known relationship with lung cancer. Consequently, there is an urgent need to understand the risk factors associated with radon exposure, and how this can be harmful to the health of exposed populations. This article presents a comprehensive review of studies indicating a correlation between indoor radon exposure and the higher probability of occurrence of health problems in exposed populations. The analyzed studies statistically justify this correlation between exposure to indoor radon and the incidence of lung diseases in regions where concentrations are particularly high. However, some studies also showed that even in situations where indoor radon concentrations are lower, can be found a tendency, albeit smaller, for the occurrence of negative impacts on lung cancer incidence. Lastly, regarding risk remediation, an analysis has been conducted and presented in two core perspectives: (i) focusing on the identification and application of corrective measures in pre-existing buildings, and (ii) focusing on the implementation of preventive measures during the project design and before construction, both focusing on mitigating negative impacts of indoor radon exposure on the health of populations.

## 1. Introduction

World Health Organization (WHO) defines radon as an inert gas released during the decay of uranium (^238^U), and which is ubiquitous in indoor and outdoor air [1]. This report refers to numerous evidence from studies documenting radon as a cause of lung cancer in smokers and nonsmokers. In fact, there are several works, more or less from the same period, referring that radon should be potentially considered an important cause of lung cancer for the general population, which is permanently exposed through the contamination of indoor air by radon emanated from the soil and water, as presented by Samet & Hornung, or by Lowry [2,3].

This potential exposure to radon entails serious health risks for those exposed, with several studies indicating that radon exposure is the second leading cause of lung cancer, which in turn is the leading cause of cancer-related deaths worldwide [4,5,6,7,8]. These studies present radon itself as a risk factor, but also because of its ability to increase the risk of lung cancer in smokers [9]. However, calling attention to the risk of radon exposure was not initially assumed to be something of immediate and direct recognition, always starting as a theme naturally related to underground miners, as referred in the work presented by Field et al., where the authors state that exposure to high concentrations of radon progeny produces lung cancer [10]. This approach does not refer yet to the designation of “indoor radon concentration” and to its associated risks, as there still does not seem to be established a relationship, in these studies, between the concentration of indoor radon and the increased incidence of lung cancer.

The relationship between radon exposure and the incidence of lung cancer appears in the articles presented, for example, by Gaskin et al. or Ruano-Gavina et al., which refer to radon being the second most important cause of lung cancer worldwide, and that lung cancer is ranked by WHO as one of the leading causes of mortality [11,12]. These authors, based on an updated database of national radon exposures for 66 countries, presented a global burden of lung cancer mortality attributable to radon, concluding that lung cancer deaths resulting from radon exposure for these countries represent the percentile of 3.0% of total cancer deaths and that the evaluation models used confirmed that residential radon is responsible for a substantial proportion of lung cancer mortality worldwide. Vogeltanz-Holm & Schwartz presented a study in which they assessed the degree of perception about whether radon can cause lung cancer [13]. The authors reviewed 20 studies, having as their starting point the question “*Have you heard about radon?*”, concluding that many segments of the population, especially younger individuals, and those with less education, do not know what radon is. Another study, presented by Lopes et al., confirms the results presented by Vogeltanz-Holm & Schwartz, through a survey of the Portuguese population, and where a significant majority of respondents say they have never heard of radon, and even those who did, presented a huge lack of knowledge of aspects related to the risk of exposure and its consequences [14]. Thus, it seems that there is still no awareness on the part of populations for the real effects of radon exposure, especially when this exposure occurs in an indoor environment not related to the mining activity, although there are already many and diverse studies that address residential radon exposure, specifically dedicated to lung cancer risk. For example, Lorenzo-Gonzalez & Ruano-Ravina accused the relationship between residential radon exposure and lung cancer risk [15]. This study, which involved 3704 people, showed that the risk of lung cancer incidence increases with radon exposure, with a significant association at radon exposures above 50 Bq·m^−3^, and that, in the case of smokers, lung cancer risk increases markedly as radon concentration increases. Also, Rodríguez-Martínez et al., addressed the relationship between residential radon exposure and lung cancer occurrence, concluding that small cell lung cancer is the incident cancer type caused by radon exposure [16]. Thus, seems justified the need to find methodologies for assessing the relationship, so often referred, between residential radon exposure and lung cancer, so that it can be easily transmitted to populations, regarding the need to prevent exposure to high radon concentration, both in residential locations and in workplaces. The main objectives of this article are to carry out a review of the historical process of the relationship between indoor radon exposure and the incidence of lung cancer, to then proceed with an analysis of the different methodologies used to establish this relationship.

## 2. Materials and Methods

To carry out this study, a systematic search was carried out using the database provided by Web of Science Core Collection (Clarivate), where the following keywords were used: “*radon*”; “*lung cancer*”; “*radon risk assessment*”; “*radon mitigation measures*”; “*natural radiation*”; “*radiation effects on health*”; “*radon effects on the environment*”; “*radon sources*”; “*radon mutagenic effects*”; “*indoor radon potential*”; “*radon detection equipment*”; “*radon current developments*”; and “*radon-induced diseases*”. Subsequently, articles were selected for their relevance, specifically, considering as the most relevant works published in journals indexed in Web of Science and Scopus, as the first selection factor, and for the number of citations for the article, as the second selection criterion. When searching the database using only the term “*radon*” as part of the title/abstract/keywords, the result obtained for the number of publications was 26,167. However, when this search is restricted to using the term “*radon*” as part of the title, the result obtained for the number of publications is substantially reduced to 13,858. In view of this difference, the search for keywords was carried out in the database always as part of the title/abstract/keywords, to avoid discarding publications that do not contain the keywords directly in the title. The obtained results are presented in Table 1.

**Figure 1:**
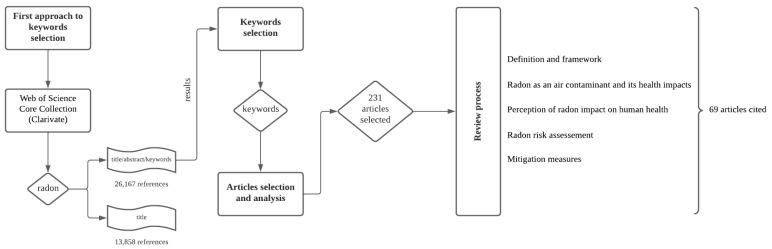
Flow diagram of the full literature screening process.

With the selected articles, the themes were reviewed in the light of the perspective that the authors have on the subject, to be able to fulfill the objective proposed for the present work, which is to establish and substantiate a relationship between the incidence of lung cancer and the exposure to high indoor radon concentrations. Figure 1 presents the flow diagram of the full literature screening process.

## 3. State-of-the Art

### 3.1. Definition and Framework

**Figure 2:**
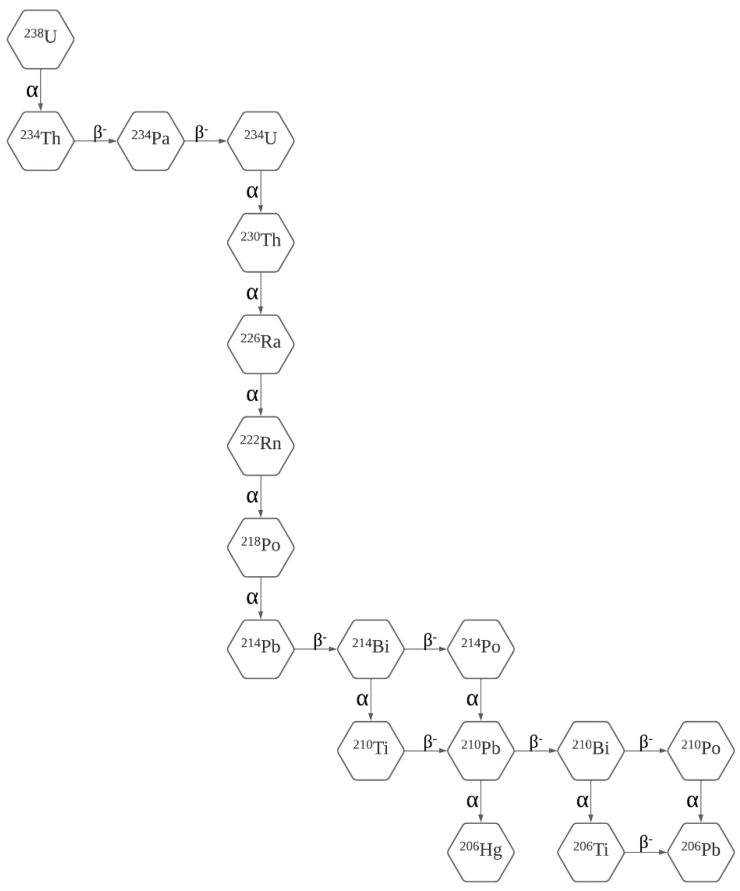
Uranium decay sequence and the radon progeny (adapted from [23,24,25]).

Radon is a noble gas that occurs naturally in soils, rocks, water, and air, which is formed through the decay of radioactive elements such as uranium [8]. It is a radioactive, colorless, odorless, and practically chemically inert gas, although it forms some compounds such as clathrates and complex fluorides [17]. Radon is soluble in cold water, and this solubility significantly decreases with increasing water temperature [18]. In fact, this feature contributes to the release of radon during hot water use processes, such as bathing, laundry, or dishwashing activities using hot water. Radon is also the heaviest of noble gases and has the highest melting point as well as the highest boiling point, the highest critical temperature, and the highest critical pressure [19]. Twenty-seven radon isotopes are known, ranging from ^200^Rn to ^226^Rn, and which have half-lives, that is, the time for the one-half of the radioactive isotopes to decay, shorter than one hour, except for ^222^Rn, ^210^Rn, and ^211^Rn, which show, respectively, 3.8 days, 2.5 h, and 14.7 h [20]. From all these radon isotopes the most relevant one is ^222^Rn, comprising approximately 80% of all radon exposure [21,22]. Figure 2 presents the uranium decay sequence and the radon progeny.

In this way, radon, being radioactive, when released into the air starts its spontaneous decay, giving rise to atoms of new elements, called radon progeny, which are electrically charged and can be associated with indoor dust particles [26]. This is precisely where the danger to human health lies, as these dust particles can be inhaled, and inside the lungs, they adhere to the mucous membranes of the lungs. These radon atoms will then have their decay process inside the human body, where they will emit a type of radiation called α (alpha) radiation, which can damage lung cells, since radiation α can damage the DNA of these cells, and if the process continues, it can lead to cancer [27]. However, radiation α can only travel very short distances inside the body, so this radiation α cannot reach other organs than the lungs [28]. Thus, most likely, lung cancer is the only potentially important cancer hazard posed by radon in indoor air [1,29,30].

### 3.2. Radon as an Air Contaminant and Its Health Impacts

Radioactive contamination, whether of natural or anthropogenic origin (usually accidental, or caused by human activity, such as the use of radioactivity or ionizing radiation for diagnostic, therapeutic purposes, or because of industrial activity or production) represents a significant hazard affecting the environment and human health [1,8]. Of all the radioactive products that can directly affect mankind and the environment, radon represents a very significant portion, being the main source of natural radioactivity to which populations are exposed. In some situations, such is the case of many locations in the United States, artificial radiation can surpass natural radiation, potentiating even more the exposed populations to the effects of natural radiation [31]. For this reason, it is also expected that the impacts on human health deriving from radon exposure are more important and consequential than those that may be caused by other sources of radiation, even occasionally, especially in the case of exceptional situations arising from causes anthropogenic events, such as accidents, this radiation can be occasionally stronger and more harmful to man and the environment [1]. For example, Bersimbaev & Bulgakova describe in their study that high radon levels can suffer from a cumulative effect if associated with long-term and large-scale mining of uranium, and where the authors relate to residential radon exposure of humans in uranium mining and milling areas in the North and East areas of Kazakhstan [32]. This radon exposure is, on average, corresponding to more than 50% of the average human dose to which populations are subject, and may also be increased by the dose resulting from other sources, which are potentially dangerous if they have the cumulative effect, contributing for harmful effects on the human body, namely and consequently carries a risk of radiation-induced cancer. If this exposure also occurs with the presence of another type of radiation, then, as verified by Bersimbaev & Bulgakova, the results indicate an increased frequency of chromosomal aberrations in blood lymphocytes, but many other types of anomalies may occur as well.

Popp et al., in the study they carried out on miners from the former German Democratic Republic, who were subjected to extremely high radiation exposures, present to be at high risk for lung carcinogenesis, with the annual incidence rates increasing significantly [33]. This study also aimed to test the range of biomarkers for DNA damage and inflammation in leukocytes and bronchoalveolar fluid for their ability to detect biological effects, having detected high frequencies of chromosomal aberrations in blood lymphocytes and an increased prevalence of both fibronectin and tumor necrosis α factor in the bronchoalveolar fluid. Thus, from the studies presented by Bersimbaev & Bulgakova and by Popp et al., which were confirmed in two studies by Darby et al., the risk of occurrence of lung cancer rises significantly if other factors, such as tobacco consumption, are cumulatively verified [8,34]. In fact, the impact of radon-related cancers worldwide, according to the United Nations Scientific Committee on the Effects of Atomic Radiation (UNSCEAR), radon can be responsible for about 20% of all lung cancer diseases, with an impact of approximately 21,000 new cases in the USA, as presented by Neri et al., and with Milner et al., reporting 1400 annual deaths for the United Kingdom, victims of radon-induced lung cancer [35].

### 3.3. Perception of Radon Impact on Human Health

Sabbarese et al., give particular importance to radon transport models, as these authors understand that they are responsible for the distribution of different concentrations in different types of dwellings, thus allowing to assess indoor radon concentration while understanding the mechanisms behind the circulation and transport of radon [36]. The authors assume that the indoor radon concentration is directly related to the construction factors, since buildings located on the same lithological and geological substrate, but with different construction factors, may also present different indoor radon concentrations. Thus, during the investigation, the authors concluded that, in addition to the nature of the soil, the position of the lower floor of dwellings plays a significant role in determining the amount of radon entry into buildings, definitively confirming the need for assessing the health hazards coming from radon accumulation in living environments.

This approach, focusing on the constructive aspects of buildings, was studied by Khan et al., in an attempt to demonstrate the impacts that the changes introduced in residential build practices have on the reduction of indoor radon concentrations [37]. For this, the authors compared long term radon tests and buildings from 25,489 Canadian and 38,596 Swedish residential properties constructed after 1945, demonstrating that the provisions in the 2010 Canada Build Code have not significantly reduced innate radon risks, highlighting the urgency of novel code interventions to achieve systemic radon reduction and cancer prevention. Additionally, the authors also concluded that the introduction of energy efficiency measures (such as heat recovery ventilation) in the respective building codes of each country are independent of radon fluctuations over time.

This concern currently focused on the risk that populations may be subject to, in fact, has been the motto for the development of works describing methodologies for the estimation of residential radon exposure, and the definition of radon priority areas based on expected lung cancer incidence, as, for example, in the study presented by Elío et al. [38]. These authors used data collected from 32,000 indoor radon measurements, collected in Ireland, with which to calculate the effective doses, which ranged within the range 0.8 to 13.3 mSv·y^−1^. Subsequently, they calculated the radon-related lung cancer incidence using the dose-effect model and obtained an incidence per million people per year between 15 and 239 cases. Based on these results, the authors estimated that of the 2300 lung cancer cases that are diagnosed annually in Ireland, around 280 may be directly related to radon exposure, concluding that although it is still necessary to refine the prediction models through the inclusion of other factors, such as tobacco consumption and other occupational diseases, the spatially presented approach defines areas with the expected highest incidence of radon-related lung cancer, even though indoor radon concentrations for these areas may be moderate or low, and recommend that both indoor radon concentration and population density by small area are considered when establishing national radon action plans.

The development of continuous monitoring equipment that enables permanent monitoring of indoor air quality parameters also began to include the assessment of radon. There are already several projects that followed this approach, such as the work presented by Alvarelhos et al., in which the authors describe the automatic low-cost air quality control system [39]. In this specific case, the system, in addition to monitoring the air quality, with radon included, determines the operation of the ventilation system, so that it can automatically correct the concentrations verified, back to the desired values. Also, in this perspective, Lopes et al. and Martins et al. presented the RnMonitor project, where they describe the development of a WebGIS-based platform for a straightforward in situ deployment of IoT edge devices and effective radon risk management [40,41]. These approaches already allow us to foresee a set of home automation integration solutions to solve existing problems, but also to be integrated even in the pre-constructive project phase of new buildings.

### 3.4. Radon Risk Assessement

**Figure 3:**
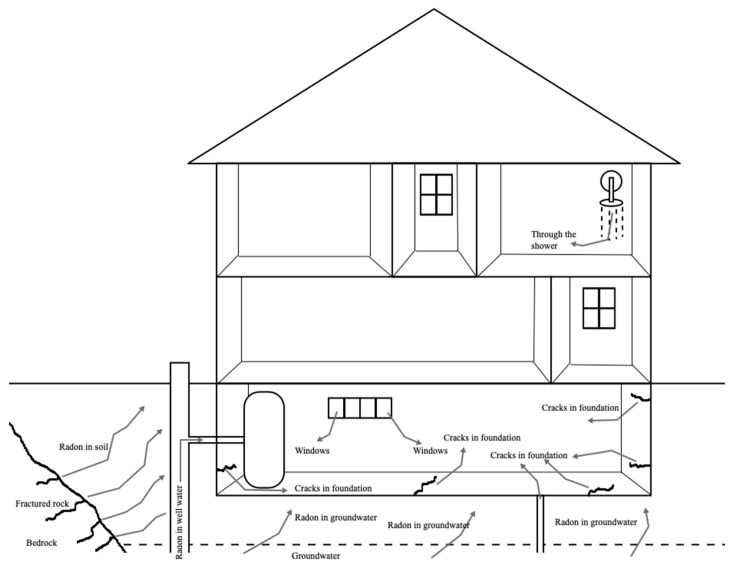
Radon can enter into buildings through cracks in the building structure after being released from rocks to soils and water. Water can transport radon into the buildings, as being highly soluble in cold water, this solubility is highly reduced in hotter water, being immediately released, for example, during showering (adapted from [43,44,45,46]).

The risk associated with indoor radon has been proven through the publication of several works, which present the results obtained in the measurements carried out inside services or intended for housing. Figure 3 shows how radon usually enter buildings. Many of these studies have shown that the risk can even be higher in residential buildings than in public buildings, with this difference being justified by the fact that the spaces’ ventilation processes contribute to aeration, and thus promote the dilution of indoor radon concentration. In fact, the procedures associated with the cleaning of public buildings, which normally follow previously defined protocols, which include the aeration of spaces, as well as the existence of HVAC systems that promote the complete renewal of the air, complying with temporary cycles, contribute significantly and effectively to reduce the concentration of indoor radon in these spaces. On the other hand, in residential buildings, this air renewal may not take place for long periods, such as during the night in the winter period, or during the day, when residents are away for work. Chen proves precisely this situation, determining, in the study carried out using data obtained from several radon surveys, that the radon progeny concentrations in Canadian homes can be up to three times higher than in school buildings, 4.7 times higher than other public buildings and indoor workplaces, and 12 times that of outdoor air [42]. This author also lists the time citizens spend in these spaces, presenting results that indicate that Canadians spend 70% of their time at home, 20% indoors away from home, and 10% outdoors. These results indicate that, due to the relatively high indoor radon concentration in residential homes, and the longer time spent indoors at home, this exposure contributes to 90% of the radon-induced lung cancer risk.

Whether this exposure occurs at home or work, what is important to mention is that, in the end, all exposure moments will contribute to the effective dose to which each person was subjected so that all efforts to assess the risk are important and contribute to the knowledge of the real conditions to which people are exposed, which is very likely the main reason that has led to such a large number of works on radon risk assessment. These studies, which initially took place in Europe and the USA, with abundant studies throughout the 21st century, quickly spread to other territories, which also began to pay attention to the theme of indoor radon concentrations, and some of them contributed interesting conclusions. For example, Teiri et al., in the study they conducted in the buildings of a university campus in central Iran, where they determined the effective dose to which the occupants are exposed, the authors also looked at the distribution of concentration on the different floors of the buildings, as well as the seasonal variability of indoor radon concentration [47]. In this specific case, the authors concluded that indoor air ventilation, the typology, and architecture of buildings, as well as construction materials, are, together with the geological structure of the ground, certainly the main factors influencing the concentration of radon inside the buildings.

The use of a dosimetric approach for effective risk assessment and subsequent management has allowed for a more integrated and comprehensive analysis, which leads to the implementation of exposure risk mitigation measures through the implementation of preventive and/or corrective measures. An example is presented in the work by Curado et al., where the authors analyze the high occupancy rate of the fan elementary school building that was retrofitted, with low energy consumption, and in the central climatization systems for heating, cooling, and ventilation [48]. The authors concluded that, based on the results, there is evidence that the risk associated with the exposure to radon gas in indoor environments does not depend only on its concentration on the monitored locations, but on the number of occupants, period of occupancy, ventilation rate, and the location of the room in the building.

### 3.5. Mitigation Measures

The implementation of mitigation measures to reduce the risk of radon exposure indoors has been the object of study interest, mainly because these measures meet the anxieties of populations, especially from the moment they become aware of the risk to which they may potentially be exposed, and, as seen in the previous section, with this exposure to define the degree of risk, following the other factors that contribute to the calculation of the exposure dose, and thus, the risk assessment [13,49,50]. Thus, there are several studies that present mitigation measures that contribute to the reduction of risk, on the one hand, but also effectively to the reduction of cases of radon-induced cancers, namely, lung cancer [51]. Within the mitigation measures proposed in the various works, these can include different types of measures, which can be divided into different groups [52]. For example, constructive measures include the application of insulation materials, such as special paints and screens, which prevent the passage of radon from the ground to indoor spaces and that can be used in the renovation of pre-existing buildings and in new buildings during the construction phase, or even the construction of airboxes between the ground and the floors/walls when possible [53,54]. On the other hand, measures related to the ventilation of indoor spaces are related to the installation of systems that renew the air, and which may or may not be associated with complex systems for continuous monitoring of air quality parameters, or simply, without permanent monitoring, protocols for manual ventilation of spaces may be implemented, with the establishment of routines for opening doors and windows to create air circulation circuits [55,56,57].

## 4. Discussion

As seen in the previous sections, exposure to indoor radon is identified by the WHO as the second cause of lung cancer, immediately after tobacco consumption, so the risk assessment of radon exposure is assumed to be a factor of concern for populations, as it has negative and significant impacts on their health. Thus, over the last decades, several methodologies have been used to somehow quantify the population risk of radon-induced lung cancer. The methodologies presented in the many works available point towards a preferential use of data obtained in radon surveys, of the grab sampling method type, on which an analysis is subsequently carried out on the number of occurrences of lung cancer within the sample for which a survey was carried out, inferring a probability of occurrence, or a percentage of cases attributable to radon. An example of a study with a methodology similar to the one described is the one presented by Chen et al., which was based on a radon survey carried out in the late 1970s in 19 cities in Canada [58]. This survey observed the radon concentration in 14,000 dwellings, with the results following a log-normal distribution, with a geometric mean of 11.2 Bq·m^−3^ and a standard deviation of 3.9. The study authors, based on the results obtained, estimated that approximately 10% of lung cancers in Canada resulted from indoor radon exposure. The study proceeded with a new survey, this time carried out in 2009, using long-term measurements, also in 14,000 homes located in different regions of Canada. The results also followed, as expected, a log-normal distribution, with a geometric mean of 41.9 Bq·m^−3^ and a standard deviation of 2.8. The theoretical estimate showed that 16% of lung cancer deaths among Canadians are attributable to indoor radon exposure. Given the results obtained, the authors concluded that there is a huge need to create a monitoring program at a government level, which brings together all stakeholders so that measures are taken to reduce the risk from indoor radon exposure.

In fact, this seems to be the major conclusion reached by all the studies, regardless of the methodologies used, as is also proven by the work carried out by Peterson et al. [59]. In this study, also carried out in Canada, in the province of Ontario, the authors calculated the population attributable risk percentage, excess lifetime risk ratio, life-years lost, the number of lung cancer deaths due to radon, and the number of deaths that could be prevented if all homes above various cut-points were effectively reduced to background levels. The results obtained an estimate that 13.6% of lung cancer deaths can be attributed to radon, in the region under study, which corresponds to 847 lung cancer deaths each year, with a very significant weight of 84% of these in ever-smokers. The authors also state that if all homes above 200 Bq·m^−3^ (which was the Canadian guideline at the time of the study) were remediated to background levels, it was estimated that 91 lung cancer deaths could be prevented yearly, and 233 if remediation was conducted to 100 Bq·m^−3^. In the end, once again, the authors pointed out the need for risk assessment as a measure to support the decision of public authorities, as a measure of allocating resources and implementing preventive measures.

Indoor spaces can concentrate significant amounts of radon gas, and in this way, increase the risk of exposure of their occupants, who, as seen so far in the previous sections, also have an increased risk of lung cancer. In fact, this exposure to indoor radon seems to be a problem on which the need to find ways of mitigation becomes evident, as several studies prove the increased risk associated with this radon exposure. A very recent study conducted by Simms et al., states categorically that younger North Americans are exposed to more radon gas due to occupancy biases within the residential built environment [60]. The authors used data collected from 18,971 Canadian households and calculated the annual particle radiation dose rates due to long-term residential radon exposure, and these rates were later analyzed as a function of occupancy. Based on this procedure, they determined that the current particle radiation dose rate to lungs is 4.08 mSv·y^−1^ from 108.2 Bq·m^−3^, with approximately 23.4% receiving 100–2655 mSv doses that are known to elevate human cancer risk. The study also revealed that homes built in the present century are mostly inhabited by younger people, who seem to be exposed to greater radiation dose rates (5.01 mSv·y^−1^), while homes built in the last century tend to be occupied by people older, where they are exposed to lower radiation dose rates (3.45–4.22 mSv·y^−1^). Thus, it appears that the most recent houses also have a greater number of children, pregnant women, and a higher number of residents in the houses, than what is found in older houses, justified by the fact that most couples young people tend to still find themselves in the process of family construction and have all their dependents in their care, while older couples, in most cases, and from a certain stage of life, have already seen their children leave from home to form their households. The authors understand that this issue is of particular importance since as a younger age-of-exposure to radon equates to greater lifetime lung cancer risk, the data collected reveal the existence of a worst-case scenario of exposure bias, which, for the authors, is a matter of great concern, since if there are cases of permanent and continuous exposure, serious future increases in radon-induced lung cancer in younger people can be expected.

This concern with the concentration of indoor radon comes precisely in the same line of thought presented in several works carried out in Spain, precisely to try to demonstrate that the risk associated with the incidence of lung cancer, although it is higher when the concentrations are much higher, continues to exist, as demonstrated by the conclusions of the work presented by Barros-Dios et al., where the authors state that even at concentrations far below the official guideline levels, radon may lead to a 2.5-fold rise in the risk of lung cancer [61]. However, this concern continues, and more recently, Ruano-Ravina et al. carried out a study in a region with a high concentration of indoor radon to analyze the correlation between esophageal cancer relative risk and the indoor radon concentration exposure, concluding that the results obtained suggest a possible effect on esophageal cancer mortality, recommending the performance of more robust epidemiological studies, of the case-control studies type [62]. In fact, it seems to be possible to refer to a growing concern with effects other than the incidence of lung cancer, since in addition to esophageal cancer, there is also a study by Barbosa-Lorenzo et al. and another one presented by Messin & Serre, where the two groups of researchers try separately to relate stomach cancer with radon exposure [63,64]. The results obtained in the studies seem, however, not to allow a direct and definitive association between these types of cancer and exposure to radon yet. Although the results are still incipient, all studies suggest that case-control or cohort studies should be performed to confirm this probable association, and that, if confirmed, additional preventive actions directed to mitigate or reduce radon exposure should be enforced. Also, the role of radon on other diseases is being studied and is a subject of current concern [65,66,67,68].

This seems to be the motto for other approaches, since, once the risks associated with exposure to indoor radon are understood, as well as the processes that lead to the accumulation of gas inside buildings, the development of methodologies that contribute to the mitigation of the problem permanently and actively. In the case of existing built spaces, the procedures allow, in a first phase, to assess the level of indoor radon concentration, leading to decision-making on the measures to be applied, and that, in a second phase, allows the monitoring of the effectiveness of the implemented measures. For example, using the methods described by Lopes et al. and developed within the scope of the RnMonitor project, which, using IoT edge devices, allows an effective radon risk management approach [40]. However, the ideal situation seems to prevent exposure in new residential spaces, which is possible if the assessment of radon potential is carried out still in the design phase, before the construction of the buildings. Thus, the use of a pre-diagnosis model, as presented by Silva et al., where, through an expeditious method, the need for further studies to confirm the radon potential indicated by the model is assessed, so that the best decisions can be taken concerning the implementation of mitigation measures that ensure the safety of the occupants of the interior spaces [69]. Mitigation of indoor radon concentration, both from a corrective and from a preventive perspective, seems to be the way forward to reduce the impact that the natural occurrence of this radioactive gas has on the health of populations.

**Figure 4:**
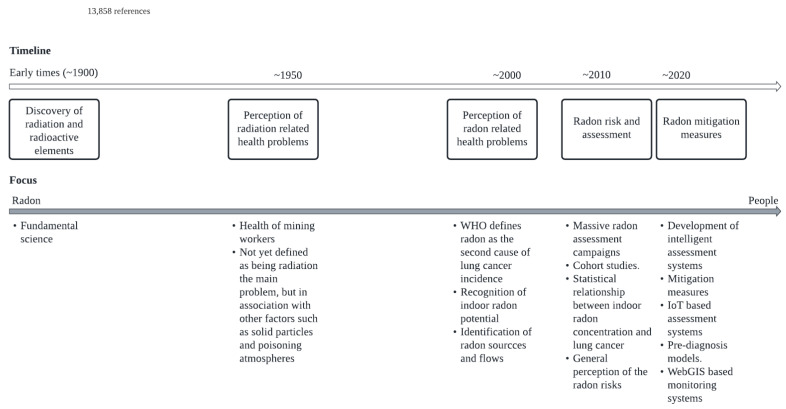
Summary of the main achievements concerning the evolution of radon science.

In this way, in the systematic review presented in this article, it is possible to perceive an evolution in the perspective of how the topic is approached. As can be seen in Figure 4, at the time of the discovery of radioactivity and radioactive elements, the focus was entirely on the knowledge about chemical elements, their characteristics, their potential for use, among others, but always within the field of fundamental science. However, with the passage of time and with the deepening of the knowledge on radon science, the focus slowly shifted to the impacts that the chemical element can have on the environment and on health, still in a mix of fundamental science and applied science. Currently, the focus is predominantly on the safety of the people and the impacts that radon has on their health, and, above all, on how these negative impacts can be minimized. In other words, the focus was completely transferred to people.

## 5. Conclusions

Radon is a noble gas naturally occurring from the decay of radioactive elements such as uranium, listed by the WHO as the second leading cause of lung cancer after tobacco. The emanation of this gas from soils and rocks into the environment does not present significant problems. However, when this release occurs in indoor and occupied environments, such as a dwelling or working places, the need to assess the risks and impacts on health is highly recommended. The prolonged use of these indoor environments contributes to the exposure of occupants to a cumulative dose of α radiation, whose negative impact depends both on the daily average indoor radon concentration and the daily average occupation period. The studies analyzed in this work reinforce this perspective while indicating the need to implement remediation measures. Many of these measures were presented in several national action plans already implemented and currently in use. These measures, e.g., the realization of national assessment campaigns that aim to identify radon occurrence potential, allow the implementation of remediation measures, such as the development of intelligent ventilation mechanisms, or the application of passive protection measures, forming a blocking barrier to radon gas, lessening the admission of radon in indoor environments.

## Figures and Tables

**Table 1:** Results obtained from the database provided by Web of Science Core Collection (Clarivate).

Keywords	Results (Nr. of Indexed Publications)
“*Radon*” + “*lung cancer*”	2659
“*Radon risk assessment*”	835
“*Radon mitigation measures*”	155
“*Radiation effects on health*”	8423
“*Radon effects on the environment*”	339
“*Radon sources*”	3003
“*Radon mutagenic effects*”	30
“*Indoor radon potential*”	514
“*Radon detection equipment*”	28
“*Radon current developments*”	77
“*Radon induced diseases*”	70

## Data Availability

The data presented in this study are available on per request to the corresponding author.

## References

[1] World Health Organization (2009). WHO Handbook on Indoor Radon: A Public Health Perspective.

[2] Samet J.M., Hornung R.W. (1990). Review of radon and lung cancer risk. Risk Anal..

[3] Lowry S. (1989). Housing and health: Indoor air quality. BMJ Br. Med. J..

[4] Sethi T.K., El-Ghamry M.N., Kloecker G.H. (2012). Radon and lung cancer. Clin. Adv. Hematol. Oncol..

[5] Zielinski J.M., Carr Z., Krewski D., Repacholi M. (2006). World Health Organization’s International Radon Project. J. Toxicol. Environ. Health Part A.

[6] World Health Organization (2007). International Radon Project: Survey on Radon Guidelines, Programmes and Activities.

[7] Ruano-Ravina A., Faraldo-Vallés M.J., Barros-Dios J.M. (2009). Is there a specific mutation of p53 gene due to radon exposure? A systematic review. Int. J. Radiat. Biol..

[8] Darby S., Hill D., Auvinen A., Barros-Dios J., Baysson H., Bochicchio F., Deo H., Falk R., Forastiere F., Hakama M. (2005). Radon in homes and risk of lung cancer: Collaborative analysis of individual data from 13 European case-control studies. BMJ.

[9] Möhner M. (2019). Re:“Reanalysis of diesel engine exhaust and lung cancer mortality in the Diesel Exhaust in Miners Study cohort using alternative exposure estimates and radon adjustment” and “diesel exhaust and lung cancer—Aftermath of becoming an IARC Group 1 carcinogen”. Am. J. Epidemiol..

[10] Field R.W., Steck D.J., Smith B.J., Brus C.P., Fisher E.L., Neuberger J.S., Platz C.E., Robinson R.A., Woolson R.F., Lynch C.F. (2000). Residential radon gas exposure and lung cancer: The Iowa Radon Lung Cancer Study. Am. J. Epidemiol..

[11] Gaskin J., Coyle D., Whyte J., Krewksi D. (2018). Global estimate of lung cancer mortality attributable to residential radon. Environ. Health Perspect..

[12] Ruano-Ravina A., Lema L.V., Talavera M.G., Gómez M.G., Muñoz S.G., Santiago-Pérez M.I., Rey-Brandariz J., Barros-Dios J., Pérez-Ríos M. (2021). Lung cancer mortality attributable to residential radon exposure in Spain and its regions. Environ. Res..

[13] Vogeltanz-Holm N., Schwartz G.G. (2018). Radon and lung cancer: What does the public really know?. J. Environ. Radioact..

[14] Lopes S.I., Nunes L.J., Curado A. (2021). Designing an Indoor Radon Risk Exposure Indicator (IRREI): An Evaluation Tool for Risk Management and Communication in the IoT Age. Int. J. Environ. Res. Public Health.

[15] Lorenzo-Gonzalez M., Ruano-Ravina A., Torres-Duran M., Kelsey K.T., Provencio M., Parente-Lamelas I., Piñeiro-Lamas M., Varela-Lema L., Perez-Rios M., Fernandez-Villar A. (2020). Lung cancer risk and residential radon exposure: A pooling of case-control studies in northwestern Spain. Environ. Res..

[16] Rodríguez-Martínez Á., Torres-Duran M., Barros-Dios J.M., Ruano-Ravina A. (2018). Residential radon and small cell lung cancer. A systematic review. Cancer Lett..

[17] Mudd G.M. (2008). Radon sources and impacts: A review of mining and non-mining issues. Rev. Environ. Sci. Bio/Technol..

[18] Schubert M., Paschke A., Lieberman E., Burnett W.C. (2012). Air–water partitioning of ^222^Rn and its dependence on water temperature and salinity. Environ. Sci. Technol..

[19] Sextro R.G. (1994). Radon and the natural environment. Radon Preval. Meas. Health Risks Control..

[20] Weigel F. (1978). Radon. Chem. Ztg.

[21] Lauria D.C. (2004). Evaluating the occurrence and spatial distribution of the radioactive isotopes 226Ra, 228Ra, 222Rn and 238U in the groundwaters of região dos lagos-rj brazil. Hidrogeologia.

[22] Pressyanov D.S., Guelev M.G., Sharkov B.G. (1995). Radon and radon progeny outdoors in a valley of enhanced natural radioactivity. Atmos. Environ..

[23] Cowart J., Burnett W. (1994). The Distribution of Uranium and Thorium Decay-Series Radionuclides in the Environment—A Review. J. Environ. Qual..

[24] Williams R.W., Gill J.B. (1989). Effects of partial melting on the uranium decay series. Geochim. Cosmochim. Acta.

[25] Ku T.-L. (1976). The uranium-series methods of age determination. Annu. Rev. Earth Planet. Sci..

[26] Wilkening M. (1990). Radon in the Environment.

[27] Robertson A., Allen J., Laney R., Curnow A. (2013). The cellular and molecular carcinogenic effects of radon exposure: A review. Int. J. Mol. Sci..

[28] Sakoda A., Ishimori Y., Yamaoka K., Kataoka T., Mitsunobu F. (2013). Absorbed doses of lungs from radon retained in airway lumens of mice and rats. Radiat. Environ. Biophys..

[29] Samet J.M., Avila-Tang E., Boffetta P., Hannan L.M., Olivo-Marston S., Thun M.J., Rudin C.M. (2009). Lung cancer in never smokers: Clinical epidemiology and environmental risk factors. Clin. Cancer Res..

[30] Tollefsen T., Gruber V., Bossew P., De Cort M. (2011). Status of the European indoor radon map. Radiat. Prot. Dosim..

[31] Ruano-Ravina A., Wakeford R. (2020). The increasing exposure of the global population to ionizing radiation. Epidemiology.

[32] Bersimbaev R.I., Bulgakova O. (2015). The health effects of radon and uranium on the population of Kazakhstan. Genes Environ..

[33] Popp W., Plappert U., Müller W.-U., Rehn B., Schneider J., Braun A., Bauer P., Vahrenholz C., Presek P., Brauksiepe A. (2000). Biomarkers of genetic damage and inflammation in blood and bronchoalveolar lavage fluid among former German uranium miners: A pilot study. Radiat. Environ. Biophys..

[34] Darby S., Hill D., Deo H., Auvinen A., Barros-Dios J.M., Baysson H., Bochicchio F., Falk R., Farchi S., Figueiras A. (2006). Residential radon and lung cancer—Detailed results of a collaborative analysis of individual data on 7148 persons with lung cancer and 14 208 persons without lung cancer from 13 epidemiologic studies in Europe. Scand. J. Work Environ. Health.

[35] Neri A., Stewart S.L., Angell W. (2013). Peer reviewed: Radon control activities for lung cancer prevention in National Comprehensive Cancer Control Program plans, 2005–2011. Prev. Chronic Dis..

[36] Sabbarese C., Ambrosino F., D’Onofrio A. (2021). Development of radon transport model in different types of dwellings to assess indoor activity concentration. J. Environ. Radioact..

[37] Khan S.M., Pearson D.D., Rönnqvist T., Nielsen M.E., Taron J.M., Goodarzi A.A. (2021). Rising Canadian and falling Swedish radon gas exposure as a consequence of 20th to 21st century residential build practices. Sci. Rep..

[38] Elío J., Crowley Q., Scanlon R., Hodgson J., Zgaga L. (2018). Estimation of residential radon exposure and definition of Radon Priority Areas based on expected lung cancer incidence. Environ. Int..

[39] Alvarellos A., Lopez Chao A., Rabuñal J.R., García-Vidaurrázaga M.D., Pazos A. (2021). Development of an Automatic Low-Cost Air Quality Control System: A Radon Application. Appl. Sci..

[40] Lopes S.I., Moreira P.M., Cruz A.M., Martins P., Pereira F., Curado A. RnMonitor: A WebGIS-based platform for expedite in situ deployment of IoT edge devices and effective Radon Risk Management. Proceedings of the 2019 IEEE International Smart Cities Conference (ISC2).

[41] Martins P., Lopes S.I., Pereira F., Curado A. RnMonitor: An IoT-Enabled Platform for Radon Risk Management in Public Buildings. Proceedings of the International Summit Smart City 360°.

[42] Chen J. (2019). Risk assessment for radon exposure in various indoor environments. Radiat. Prot. Dosim..

[43] Nero A.V., Nazaroff W. (1984). Characterising the source of radon indoors. Radiat. Prot. Dosim..

[44] Nazaroff W.W. (1992). Radon transport from soil to air. Rev. Geophys..

[45] Vaupotič J. (2002). Search for radon sources in buildings—Kindergartens. J. Environ. Radioact..

[46] Persily A.K. (1993). Modeling Radon Transport in Multistory Residential Buildings.

[47] Teiri H., Nazmara S., Abdolahnejad A., Hajizadeh Y., Amin M.M. (2021). Indoor radon measurement in buildings of a university campus in central Iran and estimation of its effective dose and health risk assessment. J. Environ. Health Sci. Eng..

[48] Curado A., Silva J.P., Lopes S.I. (2020). Radon risk assessment in a low-energy consumption school building: A dosimetric approach for effective risk management. Energy Rep..

[49] Fuente M., Rábago D., Goggins J., Fuente I., Sainz C., Foley M. (2019). Radon mitigation by soil depressurisation case study: Radon concentration and pressure field extension monitoring in a pilot house in Spain. Sci. Total Environ..

[50] Khan S.M., Gomes J., Krewski D.R. (2019). Radon interventions around the globe: A systematic review. Heliyon.

[51] Kim S.-H., Koh S.-B., Lee C.-M., Kim C., Kang D.R. (2018). Indoor radon and lung cancer: Estimation of attributable risk, disease burden, and effects of mitigation. Yonsei Med. J..

[52] Venoso G., Iacoponi A., Pratesi G., Guazzini M., Boccini L., Corbani E., Bucci S., Leonardi F., Trevisi R., Ampollini M. (2021). Impact of temporal variability of radon concentration in workplaces on the actual radon exposure during working hours. Sci. Rep..

[53] Szajerski P., Zimny A. (2020). Numerical analysis and modeling of two-loop experimental setup for measurements of radon diffusion rate through building and insulation materials. Environ. Pollut..

[54] Richter M., Horn W., Juritsch E., Klinge A., Radeljic L., Jann O. (2021). Natural Building Materials for Interior Fitting and Refurbishment—What about Indoor Emissions?. Materials.

[55] Levasseur M.-E., Poulin P., Campagna C., Leclerc J.-M. (2017). Integrated management of residential indoor air quality: A call for stakeholders in a changing climate. Int. J. Environ. Res. Public Health.

[56] D’Avino V., Pugliese M., Verde G.L. (2020). Effectiveness of passive ventilation on radon indoor level in Puglia Region according to European Directive 2013/59/EURATOM. Indoor Built Environ..

[57] Dovjak M., Virant B., Krainer A., Zavrl M.Š., Vaupotič J. (2021). Determination of optimal ventilation rates in educational environment in terms of radon dosimetry. Int. J. Hyg. Environ. Health.

[58] Chen J., Moir D., Whyte J. (2012). Canadian population risk of radon induced lung cancer: A re-assessment based on the recent cross-Canada radon survey. Radiat. Prot. Dosim..

[59] Peterson E., Aker A., Kim J., Li Y., Brand K., Copes R. (2013). Lung cancer risk from radon in Ontario, Canada: How many lung cancers can we prevent?. Cancer Causes Control.

[60] Simms J.A., Pearson D.D., Cholowsky N.L., Irvine J.L., Nielsen M.E., Jacques W.R., Taron J.M., Peters C.E., Carlson L.E., Goodarzi A.A. (2021). Younger North Americans are exposed to more radon gas due to occupancy biases within the residential built environment. Sci. Rep..

[61] Barros-Dios J.M., Barreiro M.A., Ruano-Ravina A., Figueiras A. (2002). Exposure to residential radon and lung cancer in Spain: A population-based case-control study. Am. J. Epidemiol..

[62] Ruano-Ravina A., Aragonés N., Pérez-Ríos M., López-Abente G., Barros-Dios J.M. (2014). Residential radon exposure and esophageal cancer. An ecological study from an area with high indoor radon concentration (Galicia, Spain). Int. J. Radiat. Biol..

[63] Barbosa-Lorenzo R., Barros-Dios J.M., Ruano-Ravina A. (2017). Radon and stomach cancer. Int. J. Epidemiol..

[64] Messier K.P., Serre M.L. (2017). Lung and stomach cancer associations with groundwater radon in North Carolina, USA. Int. J. Epidemiol..

[65] Kang J.-K., Seo S., Jin Y.W. (2019). Health effects of radon exposure. Yonsei Med. J..

[66] Nagy K., Berhés I., Kovács T., Kávási N., Somlai J., Kovacs L., Barna I., Bender T. (2009). Study on endocronological effects of radon speleotherapy on respiratory diseases. Int. J. Radiat. Biol..

[67] Al-Zoughool M., Krewski D. (2009). Health effects of radon: A review of the literature. Int. J. Radiat. Biol..

[68] Laurier D., Valenty M., Tirmarche M. (2001). Radon exposure and the risk of leukemia: A review of epidemiological studies. Health Phys..

[69] Silva J.P., Lopes N., Curado A., Nunes L.J.R., Lopes S.I. Designing a qualitative pre-diagnosis model for the evaluation of radon potential in indoor environments. Proceedings of the ICEER 21—8th International Conference on Energy and Environment Research.

